# YKL-40 as a biomarker in various inflammatory diseases: A review

**DOI:** 10.11613/BM.2024.010502

**Published:** 2023-12-15

**Authors:** Nina Blazevic, Dunja Rogic, Stipe Pelajic, Marijana Miler, Goran Glavcic, Valentina Ratkajec, Nikolina Vrkljan, Dejan Bakula, Davor Hrabar, Tajana Pavic

**Affiliations:** 1 Department of Gastroenterology and Hepatology, Sestre milosrdnice University Hospital Center, Zagreb, Croatia; 2 Department of Laboratory Diagnostics, University Hospital Center Zagreb, Zagreb, Croatia; 3 Department of Clinical Chemistry, Sestre milosrdnice University Hospital Center, Zagreb, Croatia; 4 Department of Surgery, Sestre milosrdnice University Hospital Center, Zagreb, Croatia; 5 Department of Gastroenterology, General Hospital Virovitica, Virovitica, Croatia; 6 Department of Internal Medicine, Intensive Care Unit, Sestre milosrdnice University Hospital Center, Zagreb, Croatia

**Keywords:** chitinases, Chitinase-3-Like Protein 1, biomarkers, YKL-40, inflammation

## Abstract

YKL-40 or Chitinase-3-Like Protein 1 (CHI3L1) is a highly conserved glycoprotein that binds heparin and chitin in a non-enzymatic manner. It is a member of the chitinase protein family 18, subfamily A, and unlike true chitinases, YKL-40 is a chitinase-like protein without enzymatic activity for chitin. Although its accurate function is yet unknown, the pattern of its expression in the normal and disease states suggests its possible engagement in apoptosis, inflammation and remodeling or degradation of the extracellular matrix. During an inflammatory response, YKL-40 is involved in a complicated interaction between host and bacteria, both promoting and attenuating immune response and potentially being served as an autoantigen in a vicious circle of autoimmunity. Based on its pathophysiology and mechanism of action, the aim of this review was to summarize research on the growing role of YKL-40 as a persuasive biomarker for inflammatory diseases’ early diagnosis, prediction and follow-up (*e.g.,* cardiovascular, gastrointestinal, endocrinological, immunological, musculoskeletal, neurological, respiratory, urinary, infectious) with detailed structural and functional background of YKL-40.

## Highlights

YKL-40 is a biomarker for inflammatory diseases’ diagnosis and prediction

YKL-40 concentration increases with age and has variations in healthy population

YKL-40 is convincing in pancreatic/liver disease, arthritis, bronchitis, and sepsis

YKL-40 is debatable in cardiovascular/neurological/renal disease, diabetes, asthma

Future larger studies and age-stratified reference intervals of YKL-40 are needed

## Introduction

YKL-40 or Chitinase-3-Like Protein 1 (CHI3L1) is a highly conserved glycoprotein, also known as human cartilage glycoprotein-39, the name that is not regularly used. The most frequent terms used in the literature are *CHI3L1* for the gene and YKL-40 for the protein it produces. The term YKL-40 is based on its molecular structure and molecular weight (*1*). YKL-40 is a chitinase-like protein and, as a member of the chitinase protein family and unlike true chitinases, it binds heparin and chitin in a non-enzymatic manner (*2*-*4*). Chitinolytic enzymes are classified into the chitinase protein families 18, 19, and 20 (*5*). YKL-40 is a member of the chitinase protein family 18, subfamily A which uses a substrate-assisted reaction mechanism. In humans, members of the chitinase protein family 18 are encoded by eight genes, seven of which are located on chromosome 1 (*5*). Several single-nucleotide polymorphisms (SNPs) in the *CHI3L1* gene are responsible for up to 23% variations in serum YKL-40 concentration in healthy population (*6*, *7*). Production and secretion of YKL-40 by different types of cells are efficiently regulated by various factors (*7*-*9*). YKL-40 synthesis begins in the neutrophils at the myelocyte-metamyelocyte stage, and at the mature level, it is packed in the granules containing lactoferrin, a target of atypical antineutrophil cytoplasmic antibodies (ANCA) (*8*). YKL-40 exerts the effect on the target cells through binding to the cell membrane receptors, mainly interleukin-13 receptor subunit alpha-2 (IL-13Rα2), consequently inducing intracellular signaling pathways important for its involvement in biological processes (*10*-*12*). YKL-40 has a crystal structure essential for its function in the physiological processes with significant connective tissue turnover (*13*, *14*). Although the accurate function of YKL-40 is yet unknown, the pattern of its expression in normal and disease states suggests its possible engagement in inflammation, apoptosis, response to antigen-/oxidant-induced injury, protection against pathogens, and remodeling or degradation of the extracellular matrix (ECM) (*8*, *15*). The detailed structural and functional background of YKL-40 is summarized in Table 1 (1-24).

**Table 1:** Structural and functional background of YKL-40

**Characteristic**		**Reference**
Molecular structure	Three N-terminal amino acids: tyrosine (Y), lysine (K) and leucine (L)	(*1*)
Molecular weight	40 kDa	(*1*)
*CHI3L1* gene location and specifications	Chromosome 1q31–1q32, it has 7498 base pairs (bp) with 10 exons and 8 kbp of the genomic DNA	(*5*)
SNPs	rs4950928, rs10399805, rs10399931, rs880633, rs1538372, rs2071579, rs946259, rs2275353	(*6*, *7*)
Enzymatic activity for chitin	No	(*3*, *4*)
Chitinase protein family membership	Family 18, subfamily A	(*5*)
Structure of chitinase protein family 18	Triose-phosphate isomerase barrel (β/α)_8_ domain; chitin insertion domain for substrate binding	(*2*, *3*, *13*, *14*)
Site of production	Macrophages, neutrophils, endothelial cells, smooth muscle cells, synoviocytes, chondrocytes, fibroblast-like cells, tumor cells	(*7*, *8*)
Production regulating factors	mRNAs, growth factors, cytokines (predominantly IL-6, stress influence, ECM changes, bacterial lipopolysaccharides)	(*7*-*9*)
Membrane receptors	IL-13Rα; transmembrane protein 219; galectin-3; CD44	(*10*, *11*)
Intracellular signaling pathways	Mitogen-activated protein kinase/extracellular signal-regulated kinase; phosphatidylinositol 3-kinase/protein kinase B; Wingless-related integration site/β-catenin; NF-κB protein complex; TGF-β1	(*10*-*12*, *16*, *17*)
Biological processes involvement	Apoptosis, inflammasome activation, inflammatory balance between type I/II helper T cells (Th1/Th2), anti-inflammatory (M2) macrophage differentiation, oxidant injury, sensitization to allergens, DC accumulation, TGF-β1 expression, ECM management, fibrosis (scarring), reinforced adhesion and invasion of bacteria, vascular remodeling, smooth muscle cell proliferation, loss of the endothelial barrier function, endothelial-mesenchymal transition	(*8*, *15*, *18*-*24*)
CHI3L1 - Chitinase-3-Like Protein 1. SNP - single nucleotide polymorphism. IL-6 - interleukin 6. ECM - extracellular matrix. IL-13Rα - interleukin-13 receptor subunit alpha-2. NF-κB - nuclear factor kappa-light-chain-enhancer of activated B cells. TGF-β1 - transforming growth factor β1. DC - dendritic cell.		

Over the past years, the scientific community has been focusing its attention on the intensified research of YKL-40, intending to detect and develop a biological marker for the early diagnosis, prediction, and follow-up, as well as a potential therapeutic agent in various inflammatory and neoplastic diseases. Based on its pathophysiology and mechanism of action, the aim of this review was to summarize research on the growing role of YKL-40 as a persuasive biomarker for inflammatory diseases’ early diagnosis, prediction and follow-up (*e.g.*, cardiovascular, gastrointestinal, endocrinological, immunological, musculoskeletal, neurological, respiratory, urinary, infectious) with detailed structural and functional background of YKL-40.

## Methods

**Figure 1:**
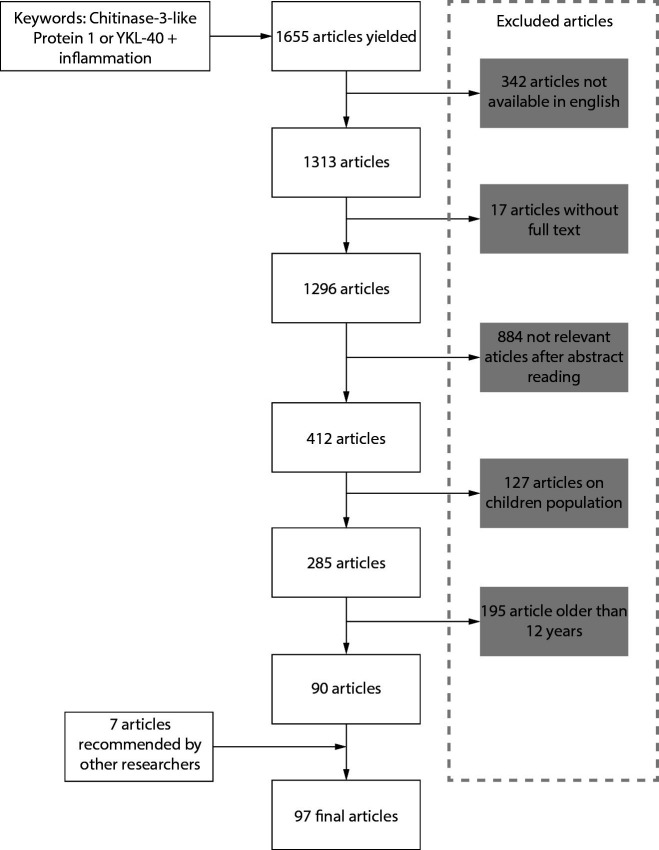
Flow chart demonstrating literature search.

A literature search was performed using the Medline/PubMed and Embase databases up to March 2023. We included human research studies published in the last twelve years (the exception is one study in inflammatory bowel disease (IBD) from 2003 and in asthma from 2008). The keywords used in the search were as follows: chitinases, Chitinase-3-Like Protein 1, YKL-40, biomarkers, and inflammation. The initial selection was performed using keywords Chitinase-3-Like Protein 1 or YKL-40 and inflammation which yielded 1655 articles. Articles that were not available in English language were excluded, which was followed with the exclusion of the articles without full text. Remain articles were screened for relevancy based on the article title and abstract, following which the full-text article was read. We included primary research articles and meta-analyses with available full text in the English language performed on cell lines or humans aged > 18 years regardless the gender. The exclusion criteria were case reports, too small group of patients based on the incidence of certain inflammatory diseases, improper statistical analysis of the data and irrelevancy to the subject which predominantly referred to the studies that investigated the role of YKL-40 in tumor diseases as this was out of the scope of this review. Finally, 7 studies were included afterwards based on the recommendation of other authors (according to the type of studies, methodology, and relevancy). The summarized flow chart demonstrating literature search is presented in Figure 1.

## Basic and animal research

The role of YKL-40 in inflammation was studied in different types of research. At the cellular level, the synovial fluid of the patients with rheumatoid arthritis (RA) is rich with neutrophils and the presence of the YKL-40 has also been found very early in RA patients (*25*). These findings led to the research on the effects of YKL-40 in various connective tissue cell cultures. In the human chondrocyte, fibroblast and synovial cell cultures YKL-40 has promotional effect on tissue growth and remodeling with an increase of the proteoglycan synthesis in a dose dependent manner (*26*). Furthermore, a dysregulation in the YKL-40 cascade may represent an important background in the development of autoimmune diseases (*27*). Although YKL-40 also has a potential autoantigenic role *via* activation of autoreactive T cells, primary Th2, animal models of iatrogenic liver injuries implicate its possible immunosuppressive ability on the function of the hepatic T cells, leading to reduced secretion of the proinflammatory cytokines and consequently preventing liver cell injury (*28*-*30*). *CHI3L1* gene expression is induced during infection, manifesting in dual action against pathogens: promotion of bacterial clearance and augmentation of host defense, and *CHI3L1* knock-out mice demonstrated more severe bacterial infection and ameliorated response to allergens (*19*, *31*). Accordingly, YKL-40 is important for the bacterial clearance and augmentation of the host defense, but it has no *in vitro* bactericidal activity (*31*).

## Laboratory methods and measurement of YKL-40

The concentration of the YKL-40 can be determined in various body specimens where different concentration can be observed in healthy controls and patients (Table 2) (*32*-*38*). Since YKL-40 is released from neutrophils during the coagulation process, concentration in serum is higher than in plasma. YKL-40 is stable for determination in the uncentrifuged plasma up to 72 hours on 4 °C, while in serum lower stability was observed (*39*). Concentrations in saliva, sputum and synovial fluid are approximately 2-3 times higher than in serum, while cerebrospinal fluid (CSF) has about 1000 times lower concentration of YKL-40 than other body fluids (*32*, *36*, *40*, *41*). The most used method for YKL-40 determination is enzyme-linked immunosorbent assay (ELISA), but recently magnetic bead fluorescent immunoassay in single or multiplex form was introduced with wider assay range and significantly shorter time to result. General characteristics of both methods are presented in Table 3 (*42*).

**Table 2:** YKL-40 concentration in different body fluids and diseases

	**YKL-40**			
**Sample type**	**Patients**	**Controls**	**Disease**	**Reference**
CSF (µg/L)	0.386 ± 0.221	0.250 ± 0.077	AD	(*32*)
Saliva (µg/L)	102.63 ± 25.85	26.27 ± 9.67	Advanced caries	(*33*)
Sputum (µg/L)	346 ± 325	125 ± 122	COPD	(*34*, *35*)
	117 ± 170	94 ± 44	Asthma	
	65.0 ± 52.9	18.7 ± 11.8	Allergic asthma	
Synovial fluid (µg/L)	237.80 ± 104.08	/	Osteoarthritis	(*36*)
Urine (µg/L)	11.75 ± 1.94	5.66 ± 3.42	AKI	(*37*)
Feces (ng/g)	1466 ± 2079	94.4 ± 215	IBD	(*38*)
Concentrations are presented as mean and standard deviation. CSF - cerebrospinal fluid. AD - Alzheimer’s disease. COPD - chronic obstructive lung disease. AKI - acute kidney injury. IBD - inflammatory bowel disease.				

**Table 3:** Characteristics of two mostly used methods for determination of YKL-40

**Method**	**ELISA**	**Bead fluorescent immunoassay**
Limit of detection (pg/mL)	3.55	3.74
Assay range (pg/mL)	78-5000	6.68-25,500
Intra-assay precision (%)	2.3-4.7	6.3-6.9
Inter-assay precision (%)	5.3-7.2	9.6-10.4
Duration of method (h)	3-5	1
Sample volume (µL)	5-10	10
Sample types	CCS, serum, plasma (EDTA/heparin), urine	CCS, serum, plasma (EDTA/heparin), urine, CSF
Required manual sample predilution	serum/plasma 1:50	serum/plasma 1:10; urine 1:2; CSF 1:100; CCS 1:5
ELISA - enzyme-linked immunosorbent assay. EDTA - ethylenediaminetetraacetic acid. CCS - cell culture supernatants. CSF - cerebrospinal fluid. Adopted from reference (*42*).		

**Figure 2:**
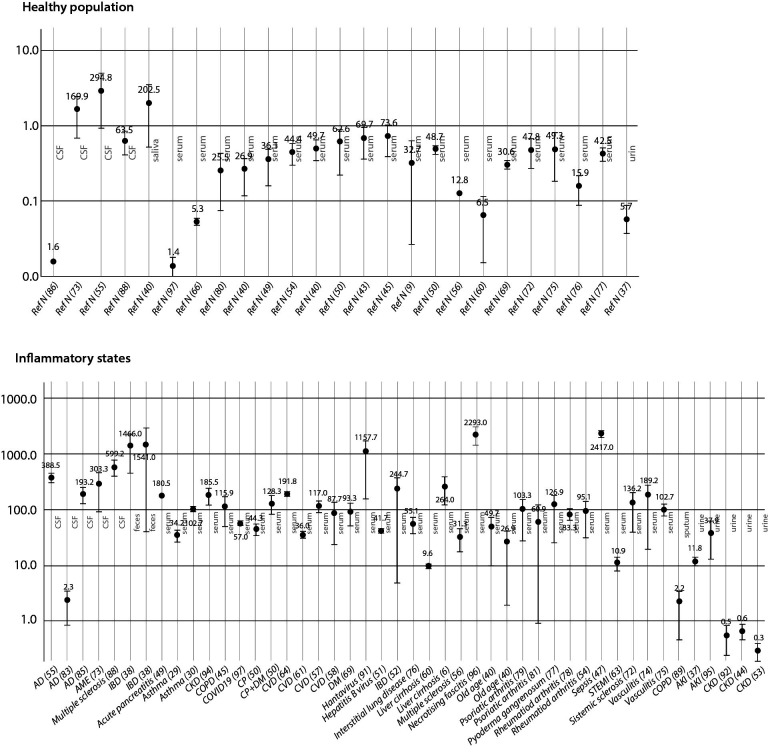
Summary of YKL-40 concentrations in reported studies including healthy population and various inflammatory states. Results of the original studies are summarized with data points representing mean concentration in µg/L (values annotated above/below points) with brackets representing standard deviation. Sample type is annotated individually. Mean and standard deviation was approximated from median and range if not reported in original study. Y-axis is log_10_ transformed for easier readability due to large range of values. Ref. N - reference number. CSF - cerebrospinal fluid. CVD - cardiovascular disease. STEMI - ST-elevation myocardial infarction. DM - diabetes mellitus. CP - chronic pancreatitis. IBD - inflammatory bowel disease. CKD - chronic kidney disease. AKI - acute kidney injury. AD - Alzheimer’s disease. AME - antibody mediated encephalitis. COPD - chronic obstructive lung disease.

The blood reference range for YKL-40 in the healthy population is still unknown due to divergent results across numerous studies. A study on 3130 healthy people in the Danish general population aged 20-80 showed a median plasma YKL-40 concentration of 40 µg/L (14-155 µg/L) that increased with age (*43*). Among older hypertensive adults with chronic kidney disease (CKD), aging (9 years interval) was associated with 13% higher concentration of urine YKL-40 compared to the baseline, more prominent tubulointerstitial inflammation, and renal repairing mechanisms (*44*). Recently published research on elderly humans found the correlation of proinflammatory cytokines in plasma and saliva (tumor necrosis factor-alpha (TNF-α), interferon-gamma, IL-6) with YKL-40, thus providing their jointed potential use as biomarkers of age-related conditions (*40*). Elevated YKL-40 concentrations have been observed in numerous inflammatory conditions, most notably asthma, chronic obstructive pulmonary disease (COPD), sepsis, diabetes mellitus (DM), acute pancreatitis (AP), chronic pancreatitis (CP), acute and chronic liver diseases, IBD, acute kidney injury (AKI), CKD, RA, Alzheimer disease (AD), multiple sclerosis (MS), and coronary artery disease (CAD). Higher serum concentrations of YKL-40 are associated with severe forms of the inflammatory diseases and consequently poorer prognosis, implicating its potential role as a biological marker in the prediction of the inflammatory response severity (*37*, *45*-*57*). Results of recent human studies in patients and healthy controls are presented in Figure 2.

## Cardiovascular system

YKL-40 was proposed as a predictor of atherosclerosis development. Due to its pathophysiologic role in embolus formation rather than in the atherosclerosis development, elevated plasma YKL-40 concentrations bear a 2-fold increased risk of ischemic stroke and venous thromboembolism, but not myocardial infarction (MI) (*18*). This finding could be explained with the involvement of YKL-40 in certain biological processes: smooth muscle cell proliferation, inhibition of pulmonary vascular endothelial cell apoptosis, inducing the loss of the endothelial barrier function, and endothelial-mesenchymal transition (*24*). In Asian population certain SNPs in *CHI3L1* gene (*e.g*. rs10399931 and rs4950928) are associated with significantly higher YKL-40 concentrations in CAD patients compared with controls, but there is no correlation with *CHI3L1* gene variants and CAD prevalence or severity (*58*). This lack of association of YKL-40 genotype and CAD risk was further corroborated by an American cohort study (*59*). A large Denmark cohort study found no complementary predictive value for the adverse cardiovascular events and mortality when YKL-40 is added to the standard predictors (*57*). These findings seem to indicate that although different genotypes can cause higher YKL-40 production in cells during inflammatory states, the molecule is not directly responsible for CAD progression. Considering YKL-40 as a diagnostic tool, in patients undergoing bypass surgery YKL-40 can increase up to 47-fold within 24 hours after surgery (36±5 µg/L and 1720±205 µg/L before and 24 hours after surgery, respectively, P < 0.001). This sudden and dramatic change not readily observed in other surgical procedures (*e.g.* liver transplant) implicates YKL-40 involvement in myocardial inflammation and fibrosis preceding myocardial revascularization (*60*-*62*). Furthermore, a Chinese study on patients with an acute MI with a ST-elevation (STEMI) demonstrated statistically significant, 2 times higher YKL-40 concentrations in STEMI patients compared to controls, arguing for YKL-40 as a potential biomarker for STEMI diagnosis (*63*). It should be noted that median YKL-40 concentrations reported in STEMI patients are lower than median YKL-40 concentrations reported in a different study on healthy elderly patients, posing a problem in terms of potential low specificity of YKL-40 as a singular biomarker in STEMI (*40*, *63*). YKL-40 has also been studied in the heart failure patients with reduced ejection fractions during chronic treatment with fixed drug combination sacubitril/valsartan. A statistically significant reduction (up to 50%) in serum YKL-40 concentration following the normalization of the heart function was observed before and 60 days after treatment, implicating its potential use as marker for evaluating treatment effectiveness (*64*).

## Digestive system

### Gastrointestinal tract

The correlation between IBD and YKL-40 concentration in serum and feces has its origin in the chronic process of mucosal inflammation and fibrosis during the disease course. Patients with active IBD have elevated YKL-40 concentrations in serum and feces compared to the controls or inactive disease (serum concentrations of 59 µg/L for both active Crohn disease (CD) (21-654 µg/L) and ulcerative colitis (UC) (26-258 µg/L) *vs.* 43 µg/L (20-124 µg/L) in healthy controls, P < 0.001), with closer correlation to disease activity in the UC than CD (*52*). Fecal YKL-40 has comparable accuracy to fecal calprotectin and can be used as a reliable biomarker of mucosal healing in IBD. When a cut-off value of 15 ng/g for fecal YKL-40 concentration was used, mean YKL-40 concentration in feces was 16 times higher in CD with endoscopic ulcerations and 12 times higher in UC with greater endoscopic subscores for disease activity, compared to CD without endoscopic ulcerations and lower endoscopic subscores for disease activity in UC (*38*). This effect is probably due to neutrophil dysregulation characteristic for IBD (especially CD) and neutrophilic origin of YKL-40. Two small cohort studies found higher prevalence of anti-YKL-40 immunoglobulin A antibodies in patients with CD compared to UC as well as healthy controls, implicating YKL-40 as a neutrophilic autoantigen in CD. Anti-YKL-40 antibodies may serve as a biomarker for CD and possibly facilitate the serological diagnosis of IBD in the near future (*28*, *65*).

### Pancreas

A possible predictive value of serum YKL-40 in the patients with an AP was observed in minor research in Denmark: on admission and 48 hours after, concentrations were 15 and 5 times higher compared with controls, respectively, in the severe forms of disease compared with milder forms (*66*). Significantly higher serum YKL-40 concentrations on admission due to assumed macrophage involvement in the peripancreatic adipose tissue were also confirmed in a more recent study (180.5±62.01 µg/L in AP *vs.* 36.1±14.14 µg/L in controls, P = 0.001) (*49*). In patients with CP investigation showed relatively low association of YKL-40 in chronic pancreatic inflammation (*50*).

### Liver

Novel biomarkers for noninvasive liver fibrosis/cirrhosis assessment, including YKL-40, are evolving, with the aim for better screening and management of patients. YKL-40 has been studied as a predictor of chronic liver disease. Three to four times higher baseline serum concentrations of YKL-40 measured in cirrhotics compared to healthy controls are associated with increased risk of alcoholic liver disease, and when used in combination with heavy alcohol drinking, calculated 10-years risk of alcoholic liver cirrhosis is up to 7% (*18*). Another study on YKL-40 in liver fibrosis assessment demonstrated that preoperative plasma concentrations of YKL-40 in the liver transplantation (LT) recipients were significantly 2 times higher than in healthy controls. YKL-40 concentrations returned to the values comparable with those in healthy controls within one day postoperatively, showing that trends in YKL-40 concentrations can be associated to the liver inflammation and fibrosis that were resolved following LT (*60*). YKL-40 has also been investigated as a prognosticator and indicator of treatment efficacy in chronic viral hepatitis. In chronic hepatitis B virus (HBV) infection in Greenlanders YKL-40 concentration greater than 200 implicated worse survival (*67*). Furthermore, in HBV infected patients, YKL-40 concentration correlated to the liver fibrosis stage with approximately 70% sensitivity and specificity for significant fibrosis prediction, superior to other noninvasive markers (*51*). Notable 20% decrease of YKL-40 serum concentration after the treatment of hepatitis C virus infection with direct antiviral agents was consistent with the improvement of other noninvasive fibrosis markers and elastography parameters, aiding in evaluation of fibrosis improvement and treatment efficacy (*68*).

## Endocrine system

YKL-40 plasma concentrations are 2-3 times higher both in patients with type 1 and type 2 DM compared to the normoglycemia group (*48*, *69*). In type 2 DM patients with concurrent obesity, YKL-40 influences insulin sensitivity through the stimulation of inflammatory chemokines production in activated adipose tissue macrophages (*69*). Patients with secondary DM (type 3) in CP have 2-3 times higher plasma YKL-40 concentrations compared to non-diabetic CP patients and healthy controls (*50*). A recent Turkish study found no association between YKL-40 and acromegaly, but discovered a significant increase in other inflammatory and immunological factors, *e.g.* C-reactive protein (CRP), advanced glycation end product and chitotriosidase (CHIT1), a marker of macrophage activation (*70*). These unexpected YKL-40 concentrations in acromegaly and CP, along with elevated concentrations of other inflammatory markers, suggest YKL-40 as a marker of yet unidentified inflammatory pattern and not as a causative agent of inflammation.

## Immune system

The role of YKL-40 has been investigated in several disorders of the immune system. In the systemic sclerosis YKL-40 concentration is higher in sputum, but not in serum, compared to healthy volunteers, regardless of the lung involvement. The underlying mechanism is in downregulation of regulatory axis that involves expression of both protein coding and non-coding RNAs that are involved in the disease development and intense local production site of YKL-40 in the lungs responsible for inflammation (*71*, *72*). Concentration of YKL-40 was elevated 2 times in CSF of the patients with an antibody-mediated encephalitis compared to healthy controls, and a cut-off value 209.5 µg/L indicates neuroinflammation and neuroaxonal injury and can aid in discrimination between cognitively normal individuals (*73*). YKL-40 has also been studied in ANCA-associated vasculitis (AAV) which demonstrated strong cytoplasmic staining of YKL-40 in inflammatory lesions and up to 2 times higher median serum concentrations of YKL-40 compared to systemic lupus erythematosus, RA, osteoarthritis and healthy controls, P < 0.001. YKL-40 may be a predictor of AAV severity with serum concentration > 220 µg/L being more frequent in severe forms, and significant reduction following AAV improvement was observed (*74*). Patients with giant cell arteritis have a correlation between circulating glycolytic enzyme pyruvate kinase M2 and YKL-40 concentrations that were significantly 2 times higher compared to controls (*75*). Serum YKL-40 concentration above 80 µg/L may be diagnostic for interstitial lung disease in an antibody-positive dermatomyositis (*76*).

## Integumentary system

YKL-40 is an inflammatory biomarker in the pyoderma gangrenosum (PG), a neutrophilic dermatosis. Although serum YKL-40 concentrations in PG are significantly higher compared to controls and YKL-40 appears to be more sensitive biomarker than other standard inflammatory markers, when putative neutrophilic origin of YKL-40 is taken into consideration, median reported concentration is lower than expected (58.4 µg/L (17.1-305.5 µg/L) in PG *vs.* 36.4 µg/L (11.1-80.0 µg/L) in healthy controls, P = 0.001) (*77*).

## Musculoskeletal system

Patients with RA have 2-2.5 times higher serum YKL-40 concentrations compared to healthy controls and positive correlation to disease activity, but together with IL-6 and vascular endothelial growth factor YKL-40 cannot predict clinical remission or radiographic progression in early RA (*54*, *78*). Recent transcriptomic data indicated that anti-TNF-α treatment suppresses the *CHI3L1* gene expression in the cells obtained from RA patients and YKL-40 might be an early indicator of non-response to anti-TNF-α treatment (*79*). In psoriatic arthritis (PsA), serum YKL-40 concentrations are 4 times increased compared to the control group (*79*). Anti-TNF-α treatment suppresses the *CHI3L1* gene expression in the cells obtained from RA patients, and significantly reduces YKL-40 concentrations in serum of PsA patients, thus providing a potential tool for monitoring the effectiveness of the anti-TNF-α molecules (*80*, *81*).

## Nervous system

The most common neuroinflammatory and neurodegenerative conditions in which the role of YKL-40 was investigated in humans are AD and MS. A longitudinal investigation of cognitively healthy individuals at risk for AD indicated an age-associated increase of YKL-40 in plasma with higher concentrations in men than women as well as positive correlation with memory (*82*). YKL-40 correlates positively with CSF sphingomyelin and galectin 3 concentrations, both molecules being key pathological biomarkers of microglial activation in AD, but surprisingly correlates negatively with brain amyloid-β deposition (*82*-*84*). YKL-40 concentration in CSF is predominantly increased in late (dementia) stages of AD with a cut-off value 316.5 µg/L to discriminate between healthy individuals (*55*). However, elevated YKL-40 concentration in CSF seems to be present regardless the etiology of neuroinflammatory disorder, such as in atherosclerosis or in previously mentioned antibody-mediated encephalitis (*73*). For instance in patients > 73 years of age, greater aortic stiffening is associated with higher concentrations of YKL-40 in the CSF, possibly due to reduced blood flow delivery to the tissue (*85*).

High serum and CSF YKL-40 concentrations are found at the beginning of the relapsing remitting MS (*56*, *86*). In a recent research, patients with MS had significantly 2 times higher serum YKL-40 concentration compared to controls (*56*). Concentration of YKL-40 in the CSF ranges between 200 µg/L and 247 µg/L, increases with the lesion load over time, and are independent of the age (*87*). Outside relapses, YKL-40 remained 18 times increased in CSF only in the patients with progressive MS (*88*).

## Respiratory system

Chronic obstructive pulmonary disease and asthma are hallmarked by the dysregulation of inflammatory processes and airway chitinases (YKL-40 and CHIT1) represent biomarkers of COPD phenotyping (*89*). YKL-40 concentrations are 2-3 times elevated both in sputum and serum of COPD patients compared to healthy controls and positively correlate with exacerbations and mortality (*45*). Serum concentrations of YKL-40 in non-eosinophilic (neutrophilic and paucigranulocytic) asthma are significantly 1.5 times higher than in eosinophilic asthma, possibly due to presumed neutrophilic origin of YKL-40 (*46*). Single nucleotide polymorphism in the *CHI3L1* gene (rs4950928) in the American Hutterite population was associated with elevated serum concentrations of YKL-40 in asthma (102.7±2.9 µg/L *vs.* 87.2 µg/L in healthy controls, P = 0.005). However, YKL-40 concentrations were not associated with the worsened lung-function (*90*). This is in accordance with above mentioned genome studies performed in CAD patients (*58*, *59*).

## Urinary system

YKL-40 in serum and urine was investigated in the AKI, CKD, and patients on hemodialysis (HD). Two times increased YKL-40 concentrations were identified both in urine and plasma of the patients with AKI compared with controls, predominantly referring to hospitalized patients or those with preexisting CKD. Plasma YKL-40 concentration was in the linear correlation to the severity of AKI and proinflammatory markers, with a cut-off concentration > 142 µg/L being predictor of adverse outcomes and mortality (*37*, *91*). In patients developing AKI as a consequence of an acute hantavirus infection, elevated plasma concentrations of YKL-40 (32 µg/L (3-213 µg/L)) persisted for a year after the hospitalization, possibly due to prolonged endothelial dysfunction (ED) after hantavirus induced vasculitis (*91*). These findings are intriguing, especially in the context of CKD where it was shown that the urine YKL-40 is an independent risk factor for the decrease in renal function and can predict development of CKD in the diabetic patients (*53*, *92*). Furthermore, diabetic patients with 2-fold increase in the plasma YKL-40 concentrations have 1.4 higher risk for HD (*93*). In patients undergoing HD, higher YKL-40 concentrations are also associated with greater risk of arteriovenous fistula patency loss (*94*). In summary, YKL-40 concentrations seem to be closely intertwined with immune mediated, infective or mechanically induced ED, thus providing a valuable research objective for early identification and improved stratification of patients in disease states etiologically associated with ED (*e.g.* CKD and CAD) (*24*, *91*, *94*, *95*). In transplanted deceased donor kidneys with AKI, increased urinary concentrations and more prominent YKL-40 expression in kidneys were associated with preferable kidney transplant recipient outcome, most likely as a part of YKL-40 upregulation in physiological response to prevent oxidative damage and activate renal repair mechanisms (*15*, *95*). In the future, there is a possibility of using YKL-40 as a valuable biomarker for the selection of donor kidneys less susceptible to ischemic or reperfusion injury following organ transplantation.

## Infectious diseases

As an acute phase reactant, YKL-40 is a tempting prognostic marker in sepsis. Several studies showed elevated YKL-40 serum concentrations in patients with sepsis compared to healthy individuals (cut-off concentration ≤ 505 µg/L is associated with better overall survival) and a positive correlation to vasopressor usage, need for HD, positive cultures, and IL-6, but not CRP concentrations. However, data demonstrated that minor allele SNP in the *CHI3L1* gene (rs4950928) leads to 5-10 times lower YKL-40 serum concentrations during severe sepsis compared to other genotypes, but with no effect on better survival. Plasma YKL-40 concentrations on admission or at the end of follow up were significantly 2-3 times higher in the patients who died in the intensive care unit compared to survivors (*47*). In patients experiencing necrotizing soft-tissue infection, higher plasma YKL-40 concentrations on admission were associated with disease severity, HD, and risk of death, but couldn’t predict a 30-day mortality (48 times higher plasma YKL-40 concentrations in dead patients compared to controls and 2 times higher compared to survivors, with a median cut-off concentration 1191 µg/L) (*96*). During the era of COVID-19 pandemic a significant linear interconnection between disease severity and YKL-40 concentrations was established. Data showed that the risk for the intensive care unit transfer increases by 0.5% for every 10 µg/L of YKL-40 concentration and plasma concentrations > 361 µg/L are indicative of a worse prognosis (*97*). The abovementioned results implicate the potential use of YKL-40 inhibitors in the treatment of COVID-19 infection in the future.

## Concluding remarks

This review represents a comprehensive and new insight into the role of YKL-40 in inflammation according to organic systems, concurrently providing important information about the structural and functional properties of YKL-40. The literature demonstrated that serum YKL-40 concentration increases with age, certain SNPs are responsible for up to 23% of variations in the serum YKL-40 concentration in healthy population, and due to divergent results across numerous studies, reference YKL-40 blood range for the healthy population is still debatable and unknown (*6*, *7*, *43*). In this review, we analysed 65 human and 32 basic and animal research studies on the role of YKL-40 as a biomarker for inflammatory diseases, with a short summary in Figure 2. Studies showed controversial results. YKL-40 can be assumed as s persuasive biomarker of diagnosis, prognosis, disease severity and activity in certain diseases, *e.g.* AP, chronic liver disease, AAV, RA, PA, COPD and sepsis. In other diseases it appears that the differences between the healthy and the patients are not particularly convincing, *e.g.* CVD, IBD, CP, DM, PG, MS, asthma, AKI and CKD. The main limitations of the studies are in small sample sizes, different YKL-40 concentrations in healthy controls, and the absence of age-stratified reference intervals for the evaluation of YKL-40 results. Moreover, the review may have excluded relevant studies due to limited access to the full-text articles and studies written in languages other than English. In conclusion, this review indicates that YKL-40 is a promising molecule with diagnostic potential in various inflammatory diseases. As a result of promiscuous expression of YKL-40 in numerous human inflammatory diseases and its non-organ-specific nature, concentrations in body fluids may vary, and careful utilization of YKL-40 as a biomarker of inflammatory disease appearance and prognosis is mandatory.

## Data Availability

No data was generated during this study.
